# Considering Exosomal miR-21 as a Biomarker for Cancer

**DOI:** 10.3390/jcm5040042

**Published:** 2016-03-29

**Authors:** Jian Shi

**Affiliations:** Department of Neurology, Department of Veterans Affairs Medical Center, San Francisco and University of California, San Francisco, CA 94121, USA; jian.shi@ucsf.edu; Tel.: +1-415-221-4810; Fax: +1-415-750-2273

**Keywords:** miR-21, cancer, biomarker, meta-analysis, sensitivity, specificity, miRNAs

## Abstract

Cancer is a fatal human disease. Early diagnosis of cancer is the most effective method to prevent cancer development and to achieve higher survival rates for patients. Many traditional diagnostic methods for cancer are still not sufficient for early, more convenient and accurate, and noninvasive diagnosis. Recently, the use of microRNAs (miRNAs), such as exosomal microRNA-21(miR-21), as potential biomarkers was widely reported. This initial systematic review analyzes the potential role of exosomal miR-21 as a general biomarker for cancers. A total of 10 studies involving 318 patients and 215 healthy controls have covered 10 types of cancers. The sensitivity and specificity of pooled studies were 75% (0.70–0.80) and 85% (0.81–0.91), with their 95% confidence intervals (CIs), while the area under the summary receiver operating characteristic curve (AUC) was 0.93. Additionally, we examined and evaluated almost all other issues about biomarkers, including cutoff points, internal controls and detection methods, from the literature. This initial meta-analysis indicates that exosomal miR-21 has a strong potential to be used as a universal biomarker to identify cancers, although as a general biomarker the case number for each cancer type is small. Based on the literature, a combination of miRNA panels and other cancer antigens, as well as a selection of appropriate internal controls, has the potential to serve as a more sensitive and accurate cancer diagnosis tool. Additional information on miR-21 would further support its use as a biomarker in cancer.

## 1. Introduction

Cancer is a major public health problem and is a leading cause of deaths worldwide. Since there is no effective treatment for advanced-stage cancer patients, the five-year survival rate may improve significantly with early diagnosis of cancer such as breast, cervical and prostate cancers. However, some cancer patients have a very poor five-year survival rate, as in the case of lung cancer where the disease is not detected until the late stages. The overall five-year survival rate of lung cancer is approximately 0%–14% [1], while the overall five-year survival rate of breast cancer at stages I and II is 100% (www.cancer.org). Therefore, it is essential to diagnose cancers in the early stages in order to improve the outcomes for patients. Over the past few decades, several biomarkers have been identified as circulating biomarkers in cancer diagnosis, including carcinoembryonic antigen (CEA) and carbohydrate antigen 19-9 (CA 19-9) [2,3]. Nevertheless, these are not capable of diagnosing most types of cancers with high accuracy, which is likely the major reason that limits their usage in cancer diagnosis.

MicroRNAs (miRNAs) are small non-coding RNA molecules of approximately 20–22 nucleotides, which post-transcriptionally regulate the production of proteins from their messenger RNAs. Their biological processes and regulatory pathways have been summarized very well in several reviews [4,5,6], and hence will not be discussed again in this review. These reviews have noted that miRNAs mediate growth, development, invasion, differentiation and progression of cancers as tumor-suppressing genes or oncogenes [7,8,9]. Additionally, miRNAs exist in several body fluids, including serum, cerebrospinal fluid, peritoneal lavage fluid, and urine, which makes them serve as robust and reproducible biomarkers for cancer diagnosis. For example, in the circulation system, miRNA levels have recently been used to identify various carcinomas [10,11]. In fact, miRNAs circulating in the serum are present in a variety of forms including within exosomes. Exosomes are 30–100 nm extracellular vesicles that are secreted from cells by exocytosis and are present in most circulating body fluids. Exosomes contain proteins, messenger RNAs and miRNAs [12]. Compared to other miRNA forms, exosomal miRNAs are more stable because they are protected from endogenous RNase degradation. Therefore, exosomal miRNAs may have significant potential as cancer-specific biomarkers.

Since the discovery of several regulatory regions of miR-21 in 2004 [13,14,15], miR-21 has been found to be over-expressed in many pathological conditions including most types of cancer analyzed so far [16]. As an oncomiR, miR-21 affects all major hallmarks of tumor-developing pathways, which include sustained proliferation through PTEN (phosphatase and tensin homolog) [17], Sprouty [18], PI3K (phosphoinositide 3-kinase) [19] and PDCD4 (tumor suppressor gene tropomyosin 4) [20,21]; impaired apoptosis through BTG2 (B-cell translocation gene 2) [22], FasL (pro-apoptotic FAS ligand) [23], FBXO11 (a member of the F-box subfamily 1) [24], and TIMP3 (inhibitor of metalloproteinases 3) [25]; and angiogenesis and invasion through PTEN [26], TIMP3 [27], and TPM1 (tropomyosin 1) [28], as well as some other pathways related to inflammation and genetic instability [29]. Importantly, a large number of studies have explored the function of miR-21 as a biomarker for cancer diagnosis. While several studies have published meta-analyses on this topic [10,11,16,30], an exosomal miR-21 meta-analysis has not been evaluated yet. In this systematic review, we perform the initial meta-analysis for exosomal miR-21 and discuss major issues related to the use of miR-21 as a potential biomarker for cancer.

## 2. Materials and Methods

### 2.1. Search Formula

For the literature search, we used two search formulas: (1) (“exosomal”) AND (“miR-21” OR “miRNA-21”) AND (“biomarker”) AND (“cancer” OR “tumor”); (2) (“miR-21” OR “miRNA-21”) AND (“biomarker”) AND (“cancer”). We performed a literature search for relevant studies using the following databases: PubMed, CNKI, and Web of Science (updated to July 13 2015).

### 2.2. Inclusion Criteria and Data Extraction

We chose studies that met the following criteria: (1) investigated the diagnostic potential of exosomal miR-21 for human cancers; (2) used the gold standard to confirm the diagnosis of cancer patients; and (3) provided sufficient data to construct a diagnostic 2 × 2 table. This table contains true positives (TP), false positives (FP), false negatives (FN), and true negatives (TN). Conversely, studies were excluded if they (1) were obviously not related to our topic or focused on other miRNAs; (2) did not have enough data to construct the diagnostic 2 × 2 table; (3) were in the forms of letters, editorials, case reports, or reviews; and (4) used types of samples other than exosomes or extracellular vesicle (EV).

### 2.3. Statistics Analysis

The SAS software (SAS Institute Inc. Cary, NC, USA) and SigmaPlot (11.0) were performed to analyze the statistics. We calculated the pooled sensitivity (TP/(TP + FN)), specificity (TN/(TN + FP)) and 95% confidence intervals (CIs) using the bivariate regression model [31]. Based on the sensitivity and specificity of eligible studies, we constructed summary receiver operating characteristic (SROC) curves by using the Moses’ fixed effects method [32]; meanwhile, the corresponding area under the SROC curve (AUC—area under the curve) was calculated.

## 3. Results

### 3.1. Literature Search Results and Summary of Studies

**Figure 1:**
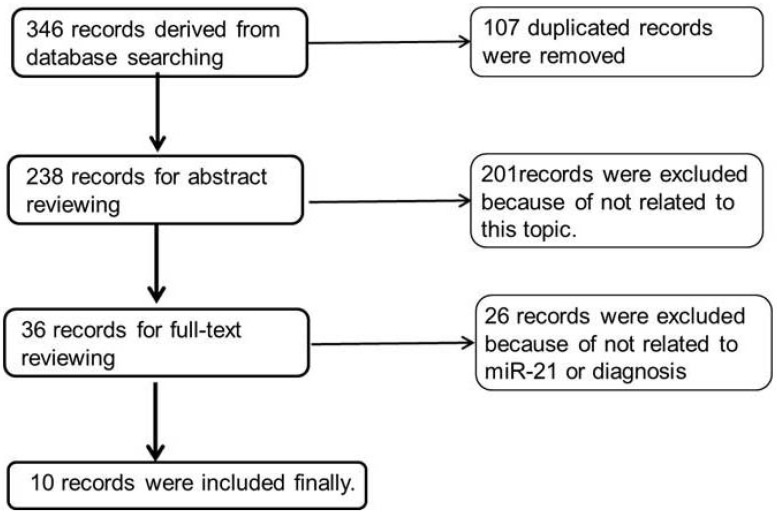
Flow diagram of the literature search process.

Only 10 of 346 records were selected [33,34,35,36,37,38,39,40,41,42] after the primary, secondary and tertiary searches following the strategy shown in the methods. Figure 1 shows the search process. Because the studies of exosomal miRNAs are in the initial stages, we used more general keywords in the second formula in order to obtain more studies. In the primary search, 346 records were gained, and 107 were excluded for duplicates and reviews after careful reading of all those titles and abstracts. In the secondary search, 201 records were excluded because they were neither related to the diagnostic study nor related to miR-21. Two records were excluded because of the unavailability of full articles. In the tertiary search, 26 records were excluded after reading 36 full articles because of the lack of data for the construction of 2 × 2 tables or the absence of exosomal miR-21 data. Finally, only 10 records were related to our topic, in which seven exosomes were from blood, two exosomes were from peritoneal lavage fluid (PLF) [42] or cervicovaginal lavage specimens (CLF) [40], and one extracellular vesicle (EV) was from the cerebrospinal fluid (CSF) [41].

Table 1 summarized the characteristics of studies that we obtained from the search process. We used the data to perform initial meta-analysis for exosomal miR-21 as a biomarker for cancer. From 2008 to 2015, these studies covered 215 non-cancer controls and 318 cancer patients. Cancers included laryngeal squamous cell carcinoma (LSCC), hepatocellular cancer (HCC), esophageal squamous cell carcinoma (ESCC), colorectal cancer (CC), gastric cancer (GC), ovarian cancer (OC), breast cancer (BC), pancreatic adenocarcinoma (PC), cervical cancer, and glioblastoma. Among these studies, seven exosomal miR-21 samples were from serum, one EV miR-21 was from CSF, and two exosomal miR-21 samples were from cervicovaginal lavage specimens (CLF) and peritoneal lavage fluid (PLF). All information from these studies is listed in Table 1, including the numbers of patients and controls, the types of cancer and sample, and 2 × 2 tables.

### 3.2. The Sensitivity and Specificity of Pooled Studies

**Figure 2:**
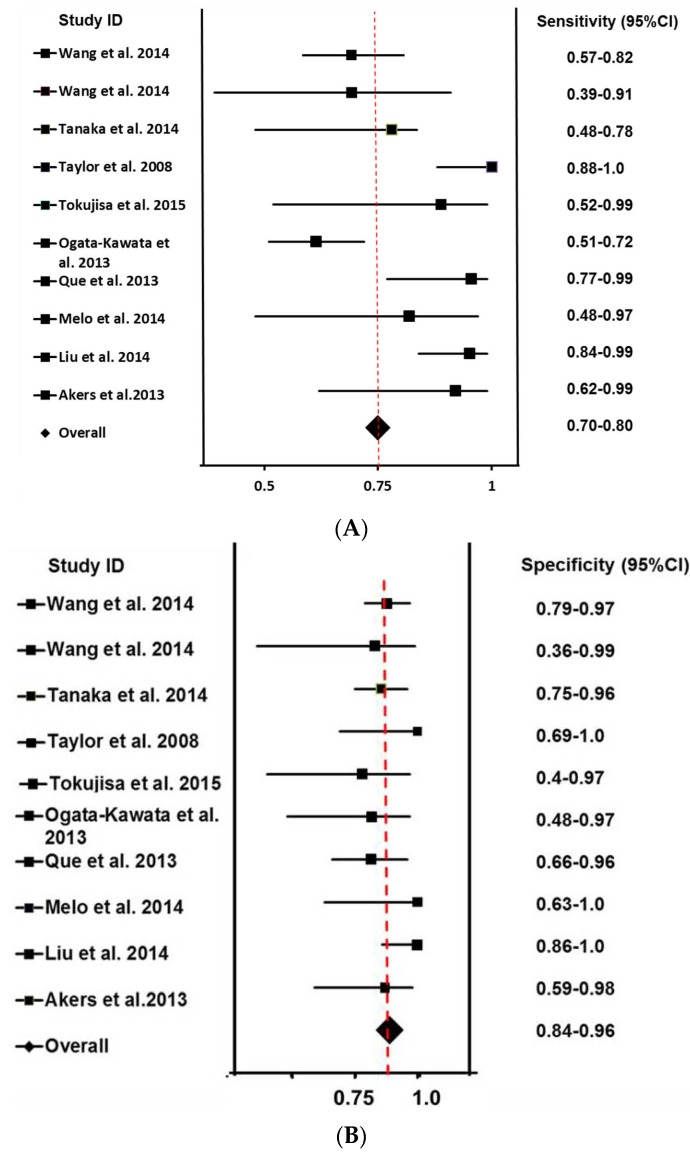
The forest plots of sensitivity (**A**) and specificity (**B**) of each included studies. In each picture, the left side shows the ID of studies, and the right side shows their 95% CIs.

Sensitivity and specificity are the most important and widely used statistic indexes for a diagnostic test. It is widely accepted that the sensitivity of a test is its true positive response, and the specificity is its true negative response. As calculated by the bivariate meta-analysis, the overall sensitivity and specificity of these studies were 75% (0.70–0.80) and 85% (0.81–0.91) with 95% CIs. Figure 2A shows the forest plot of sensitivities of all included studies and the overall sensitivity. The red line represents overall sensitivity with hepatocellular cancer (HCC) [32], CC [34] and LSCC [33] studies on the left of the red line, indicating that their sensitivities were less than 75%, while other studies are on the right. In the HCC study, 13 patients in advanced tumor stages (III and IV) and 30 healthy volunteers as controls were included with high expression of miR-21 as the cutoff point [33]. In the ESCC study, there were two groups, which were difficult to combine. One group of 44 patients and 41 controls was included with 0.02-fold as the cutoff point [37]. The gastric cancer (GC) study had no healthy control, and thus, patients at stage T1-2 were used as controls for patients at stage T3–4 [42]. In the breast cancer (BC) study, after exosomes were harvested from the serum of healthy controls and breast cancer patients, they were left in cell-free culture conditions for 24 h or 72 h, followed by qPCR being performed for all samples at the two time points. The fold-change of exosome miR-21 at 72 h was quantified relative to the exosomal miRNA at 24 h [39]. We chose 1.5-fold as the cutoff points in this study.

Figure 2B shows the specificities of all included studies as well as the overall specificity. Notably, the specificity of the GC study [42] is on the left of the red line, which is the overall specificity position. The specificities of several other studies are also on the left of the red line, though close to it.

### 3.3. The SROC and AUC of Pooled Studies

**Figure 3:**
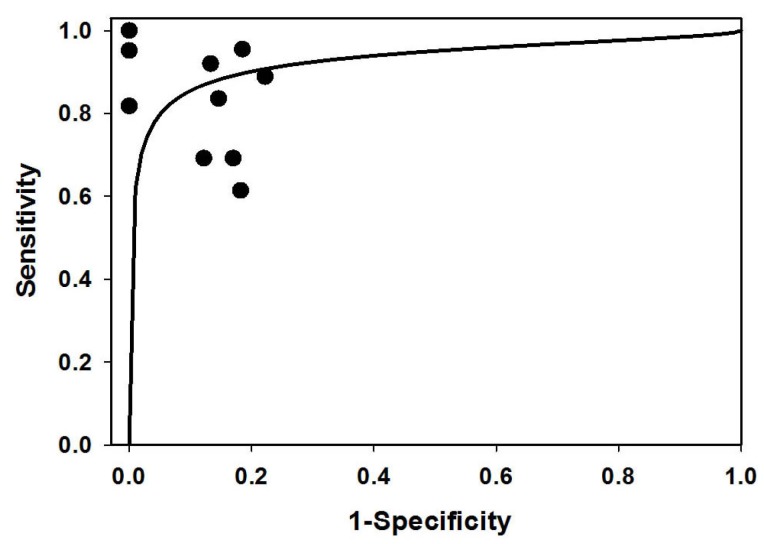
The SROC curve for different cancers with pooled studies of sensitivity and specificity. Exosomal miR-21 yielded an area under the SROC curve (AUC) of 0.93 with an overall sensitivity of 75% (0.70–0.80) and specificity of 85% (0.81–0.91) with their 95% CIs.

In 1993, Moses *et al.* [32] developed the summary receiver operating characteristic (SROC) curve, which is used to summarize the results from several independent studies for the same biomarker or the same test. In fact, the ROC curve represents a diagnostic test’s sensitivity *versus* its false positive rate (1-specificity). Although SROC and ROC curves are both plotted with sensitivity and 1-specificity, they are very different. The points of a ROC curve are usually obtained from a single study by changing the cutoff points continually, while the points of a SROC curve are from independent studies, and each point represents one study. After two decades of development, while there are more complex models for obtaining SROC curves to summarize independent studies, most curves are similar to the curve from Moses’ model [43]. Therefore, to generate a SROC curve, Moses’ model is still the most popular model. Following the process of Moses’ model, in the first fitting process, two points were outliers. To keep HCC data, we chose HBsAg negative people (FP = 1, TN = 5) as controls from all healthy controls in this study [33]. In the second fitting process, only one data point was an outlier. Figure 3 shows the SROC curve of exosomal miR-21 fitted for this study, and those dots represent all pooled studies. The area under the curve (AUC) represents diagnostic accuracy. For the SROC of included studies, the AUC was 0.93, indicating a high level of diagnostic accuracy and the possibility of using exosomal miR-21 as an overall diagnostic biomarker for cancer.

### 3.4. The Cutoff Values and Endogenous Controls

To consider exosomal miR-2 as a biomarker, we have to evaluate its cutoff value and endogenous control for cancer diagnosis. Although these studies used different methods for the isolation of exosomes, performing qPCR as well as internal controls, we could obtain pertinent information that would allow us to understand the characteristics of exosomal miR-21 as a biomarker for cancers in the future. In Table 2, we compare the cutoff values and internal controls from all studies, especially those five studies that used serum exosomal miR-21 and measured miR-21 by real-time PCR. The cutoff values were calculated using either the −ΔΔCT equation or the 2^−ΔΔCT^ equation. The methods of exosome isolation and qPCR performance are also shown in Table 2.

Usually, the 2^−ΔΔCT^ formula is calculated for the quantification of miRNA expressions, where CT is the cycle threshold and ΔΔCT = ((CTmiRNA)tumor − (CTcontrol)tumor) − ((CTmiRNA)normal − (CTcontrol)normal). The values of ΔΔCT directly indicate fold changes, while 2^−ΔΔCT^ indicates the changes in miRNA expression. In Table 2, the cutoff values without stars are original data from the listed studies, while the other cutoff points are those calculated by authors for easy comparison.

According to the upper five data points of Table 2, the cutoff point of exosomal miR-21 as a general biomarker may be close to 4.0 to 5.5 folds, between which the variation is very narrow, at least in serum-derived exosomal miR-21. The data could be compared because the studies used relative RT-PCR with all exosomes derived from blood. In the GC study, the 3.5-fold cutoff value is close to the range of 4.0 to 5.5 folds because relative RT-PCR methods were used, although the exosomes were derived from PLF and not from blood. In fact, differences in reagents for qPCR, internal controls and methods of exosome isolation would also influence the results of qPCR and the cutoff values. Therefore, it may be possible to obtain a universal cutoff point for the miR-21 in order to detect cancers after using standard reagent, internal controls, and methods and resources for the isolation of exosomes. Indeed, it is essential to obtain a universal cutoff point in order to establish a universal biomarker.

However, the cutoff values cannot be compared to each other upon using different experimental methods in the lower four rows of Table 2. For example, the ovarian cancer (OC) study used microarray analysis, whereas the BC study used values of miR-21 at 72 h *vs.* 24 h as the cutoff points. Their cutoff points had different meanings, so they could not be compared to each other. Likely, the absolute RT-PCR was performed in a glioblastoma study, while a relative RT-PCR without any internal control was used in the cervical cancer study. Although these two studies did not have internal controls, the cutoff values could not be compared because of the same reason. The cutoff value was a copy number per exosome in the glioblastoma study, whereas the cutoff value was relative folds in the cervical cancer study.

## 4. Discussion

### 4.1. The Exosomal miR-21 as a Universal Biomarker for Diagnostic Cancers

This initial meta-analysis, including 10 types of cancer, suggests that exosomal miR-21 may be a universal fluid biomarker for cancers. Compared to CA19-9 and CEA, which have widely been used as tumor biomarkers for detecting many types of cancer, exosomal miR-21 seems to be more accurate for the diagnosis of cancers. For example, the sensitivities of CEA, CA 19-9 and miR-21 for detecting colorectal cancer were 30.7%, 15.9% and 61.4%, respectively [35]. Additionally, the accuracy of exosomal miR-21 (AUC = 93) for diagnosis was likely to be better than that of circulating miR-21 in various carcinomas (AUC = 87) [10], lung cancer (AUC = 81) [1], non-small cell lung cancers (AUC = 0.775) [44], and all cancers (AUC = 88) [30]. However, we need to note that all of these meta-analyses were based on larger case numbers compared to our study. Therefore, higher patient numbers are needed for exosomal miR-21 to be compared and to confirm whether or not exosomal miR-21 is better than circulating miR-21. Importantly, several studies indicated that miR-21 may be an important regulatory molecule in carcinogenesis, and suggested that miR-21 may become a universal serum biomarker for carcinomas [36]. Conversely, there is still a lack of effective biomarkers for the early diagnosis of brain tumors even though there are many new advances in the understanding of the molecular pathogenesis of brain tumors. A miR-21 meta-analysis of brain tumors may explore the prognostic role of miR-21 expression in patients with brain tumors, in which miR-21 may be expressed in the tumor tissue or blood of patients. It has been suggested that high expression of miR-21 is associated with poor prognosis in patients with brain tumors [45]. Nevertheless, the expression levels of CSF-derived EV miR-21 from glioblastoma patients were 10-fold higher than those derived from non-tumor patients; the detected sensitivity, specificity, and accuracy were 87%, 93% and 91% (AUC = 0.91), respectively. In contrast, no significant differences in EV miR-21 levels could be detected between patients with or without glioblastoma when EVs were isolated from sera [41].

However, as an overall early biomarker, miR-21 is not appropriate for use in diagnosis of some cancers. For example, we can see in the HCC study that exosomal miR-21 could not detect HCC in stage I and II and it represented sensitivity not only in HCC patients but also in HBsAg-positive healthy controls [33]. Similarly, given that exosomal miR-21 expression increased in HPV-positive controls as well as in cervical cancer patients [40], HPV-positive patients could not be used as controls. Therefore, because the function of exosomal miR-21 in a number of diseases other than cancer is unclear, its usage is limited to being a general biomarker for cancers. In breast cancer, exosomal miR-21 was also more sensitive to the later stages of cancer [46]. Thus, some studies can use miR-21 as a progressive indicator.

### 4.2. The Combination of miRNA Panels and Cancer Antigens

Some combinations of miRNAs and cancer antigens may enhance the sensitivity and specificity of these biomarker panels for cancer diagnosis. In this regard, a combination of miR-21, miR-210 and miR-486-5p might be used to diagnose lung tumors because the miRNA panel distinguished lung tumors from benign pulmonary nodules, with the AUC of 0.86, sensitivity of 75% and specificity of 85% [47]. Additionally, another panel of plasma miRNAs, including miR-122, miR-192, miR-21, miR-223, miR-26a, miR-27a, and miR-801, provided high diagnostic accuracy (AUC = 0.89) to identify hepatocellular carcinoma (HCC) [48]. Moreover, a combination of miR-10b and miR-373 indicated that patients with lymph node–positive breast cancer, compared to those with node-negative breast cancer, had an enhanced diagnostic sensitivity and specificity up to 72% and 94%, which was better than miR-10b or miR-373 individual diagnosis [49]. In fact, this might be very helpful for surgeons to choose a breast-conserving surgery for patients with lymph node–negative breast cancer, which could greatly improve the quality of life in the post-surgery patients. Further, the aberrant serum levels of miRNAs in the panel, namely miR-21 (high), miR-126 (low), miR-155 (high), miR-199a (low) and miR-335 (low), could identify breast cancer with hormone-negative receptor status [46], in which the three common hormone receptors are not present in cancer tissues, so they are also called triple negative breast cancer. Mostly, triple negative breast cancer is more aggressive and difficult to treat, but in earlier stages, the cancer can respond to chemotherapy even better compared to other forms of breast cancers (www.nationalbreastcancer.org). Thus, this complex miRNA panel may have potential for diagnosing triple negative breast cancer in the earlier stages, and therefore, it may have potential to increase the five-year survival rate.

Furthermore, from the above example, we observed that if CEA and miR-21 were combined, the sensitivity would be 72.7%, which is better than 30.7% (CEA) or 61.4% (miR-21) individually [35]. In a pancreatic cancer study, the combination of miR-16, miR-196a and CA19-9 was even more effective in diagnosing the disease, with AUC, sensitivity, and specificity up to 0.98, 92% and 96%, respectively [50]. This study also showed that the AUC of miR-21 was 0.83, but the combination of miR-21 with CA19-9 did not significantly improve the AUC. Therefore, to achieve an effective diagnosis, not only do we need to use the combination of some miRNA panels and/or cancer antigens, but we also need to compare and choose the right combination of miRNAs and cancer antigens.

### 4.3. Choosing the Right Internal Controls

The endogenous control is very important for the normalization, reliability and reproducibility of diagnostic results because it helps to normalize differences among sample qualities and variations in detecting processes, including RNA extraction, reverse transcription procedures and reverse transcription quantitative PCR (RT-qPCR). In the pooled studies, internal controls included miR-16, U6 and miR-451, of which U6 and miR-16 seemed more popular. Recently, Xiang *et al.* compared the expressions of U6, miR-16 and miR-24 in serum following several freeze-thaw cycles, knowing that U6 expression gradually decreased after several cycles of freezing and thawing. In contrast, the expression of miR-16 and miR-24 remained relatively stable. This suggests that U6 is not an ideal internal control if freeze-thawing is required [51]. Additionally, U6 varied significantly from −1.03 to 8.12-fold in different tumors [52], and the expression levels of U6 showed a high degree of variability between the carcinoma tissues of the liver and the adjacent normal tissues [53]. Therefore, when selecting U6 as an internal control for evaluating profiles of miRNAs in freeze-thaw procedures as well as in carcinoma patients, we need to pay more attention.

Conversely, miR-16 is frequently used as a control because it is highly expressive and relatively invariant across large numbers of samples and tissues [54]; however, elevated levels of miR-16 were found in serum correlating with bone metastasis in breast cancer patients [55]. Additionally, in a pancreatic cancer study we discussed above, the combination of miR-16 and miR-24 was even used to diagnose this cancer [47]. Therefore, further investigation is required for selecting an internal normalization procedure in this scenario.

Recently, there have been several better choices for internal controls. Like miRNA panels, a panel of miR-16 and miR-425 was suggested as an internal control panel because of its more stable expressions in different cancers and controls that may lead to more accurate detection of altered target miRNA expression [56]. Conversely, for pancreatic ductal adenocarcinoma diagnostics, the internal control U91 was better than U6 or miR-16, but the most stable panel of internal controls was a combination of U6 and U91, compared to U6, miR-16 or U91 separately [52]. In addition, as internal controls of plasma miRNA research, the panel of U6 and miR-520d-5p was the best candidate after being compared with Let-7a, Let-7d, Let-7g, miR-16, U6, RNU48, miR-191, miR-223, miR-484, and miR-520d-5p. Accordingly, this control panel represented the consistency and high Ct in all studied samples and a very narrow and reproducible SD [57].

### 4.4. Comparison of Exosomal miR-21 Expression in Other Diseases

Understanding exosomal miR-21 expression in other diseases (non-cancer) would improve its use as a biomarker to distinguish cancers from other diseases. For example, the miR-21 expressed in HBsAg-positive people and live cirrhosis patients had very high true positive (TP) values, 45.8% and 52.2%, respectively, using the same cutoff points of HCC. Thus, we should treat the positive data very carefully when using miR-21 to detect HCC (69.2% TP) [33] in these people and patients. Additionally, we could not use these people and patients as negative controls when studying cancer diagnosis. Additionally, as discussed above, exosomal miR-21 expressed in HPV-positive people had 52% TP using the cutoff value of cervical cancer [40]; in fact, almost all CC cases are caused by HPV, so we should know that some people have a high expression of miR-21 and HPV positivity without cancer. In this situation, detecting high-risk HPV expression may be a useful method to distinguish some cervical cancer patients and people with a high risk of cervical cancer from regular HPV-positive people. Moreover, patents with vocal cord polyps or cholecystolithiasis were used as negative controls for the miR-21 detection of LSCC [34] or ESCC [37], knowing that exosomal miR-21 expression was lower in those patients. Further, a recent study showed that exosomal miR-21 was highly upregulated in the CSF of Japanese Encephalitis Virus (JEV) patients, in which the detected difference was approximately five folds between JEV-positive patients and JEV-negative controls using the RT-PCR method and miR-93, miR-24 and miR-103 as internal controls [58]. As shown above, exosomal miR-21 was highly upregulated (10-fold) in glioblastoma [45], but the cutoff values could not be compared here because their meanings were different. Even so, the upregulation of miR-21 in JEV patients may allow doctors to pay more attention to the false positive results generated by exosomal miR-21 when detecting glioblastoma in the virus-infected areas. Therefore, knowing more about miR-21 expression in other diseases may provide more supportive data for miR-21 use.

## 5. Conclusions and Prospect

Based on the accuracy of this initial meta-analysis and very close cutoff values from different experimental conditions, methods and internal controls, exosomal miR-21 has a very strong potential to be a good general biomarker for cancer diagnosis. The results of exosomal miR-21 seemed to be better than those of circulating miR-21 according to several comparisons we discussed above, but we know the case number was too small to give a very strong conclusion. Although the sample size was small in this study, determining and understanding the characteristics of different candidates as biomarkers for cancers are important topics in the development of the best diagnostic methods for early stages of cancers in order to increase the survival rates of the patients.

Prospectively, combining the right miRNA panels or cancer antigens may give better diagnostic results or prognostic predictions in most circumstances. To make a better diagnosis, we should also pay more attention to choosing correct, stable and consistent internal controls or internal control panels. For a general biomarker, other issues, including qPCR reagents and exosome-isolating methods, also need to be standardized. In addition, an in-depth study of various diseases for miR-21 would allow better application for cancer diagnosis.

## Figures and Tables

**Table 1:** Characteristics of diagnostic clinical studies included in this analysis.

Study ID	Patients	Controls	Cancer	Specimen	2 × 2 Table			
					TP	FN	FP	TN
Wang 2014 [33]	52	49	LSCC	Serum exosome	36	16	6	43
Wang 2014 [34]	13	30	HCC (III-IV)	Serum exosome	9	4	12	18
Tanaka 2013 [37]	44	41	ESCC	Serum exosome	28	16	6	35
Taylor 2008 [38]	30	10	OC	Serum exosome	30	0	0	10
Tokuhisa 2015 [42]	9	9	GC	PLF exosome	8	1	2	7
Ogata-Kawata 2013 [35]	88	11	CC	Serum exosome	54	34	2	9
Que 2013 [36]	22	27	PC	Serum exosome	21	1	5	22
Liu 2014 [40]	45	25	Cervical cancer	CLF exosome	40	5	0	25
Melo 2014 [39]	11	8	BC	Serum exosome	9	2	0	8
Akers 2013 [41]	13	14	Glioblastoma	CSF-EV	11	2	1	13

TP: true positives, FP: false positives, FN: false negatives, TN: true negatives.

**Table 2:** The cutoff value and internal control of exosomal miR-21.

Cancer	Cutoff Point		Internal Control	qPCR	Exosome Isolation
	Fold	2^−ΔΔCT^			
HCC [33]	5	0.03 **	U6	SYBR Green	Reagent (Life Tech.)
PC [36]	4.05	0.06 **	U6	TaqMan	Ultracentrifugation
ESCC [37]	5.66 *	0.02	miR-16	TaqMan	ExoQuick (SBI)
LSCC [34]	4.55 *	0.043	U6	SYBR Green	ExoQuick (SBI)
CC [35]	6.56 *	0.0108	miR-451	TaqMan	Ultracentrifugation
GC [42]	3.5 *	0.088	miR-16	TagMan	Ultracentrifugation
OC [38]	11 intensity	None	microarry	None	MACS
Cervical C. [40] cancer [40]	3.0-fold	None	None	TaqMan	Ultracentrifugation
Glioblastoma [41]	0.25/EV	None	None	absolute	Ultracentrifugation

MACS: magnetic activated cell sorting; * The cutoff values were calculated using ΔΔCT (patients-controls); ** The cutoff values were calculated using the 2^−ΔΔCT^ equation and then normalized to given internal control.
